# Connecting through ISEV's developing social media landscape

**DOI:** 10.1002/jev2.12475

**Published:** 2024-07-08

**Authors:** Tom. A. P. Driedonks, Deborah C. I. Goberdhan, Sujata Mohanty, Sarah Williams, Rienk Nieuwland, Kenneth W. Witwer, Ana Claudia Torrecilhas

**Affiliations:** ^1^ Department of CDL Research University Medical Center Utrecht Utrecht The Netherlands; ^2^ Nuffield Department of Women's and Reproductive Health University of Oxford, Women's Centre, John Radcliffe Hospital Oxford UK; ^3^ Stem Cell Facility, DBT‐Center of Excellence for Stem Cell Research All India Institute of Medical Sciences New Delhi India; ^4^ International Society for Extracellular Vesicles Baltimore Maryland USA; ^5^ Laboratory of Experimental Clinical Chemistry Amsterdam UMC location University of Amsterdam Amsterdam The Netherlands; ^6^ Department of Molecular and Comparative Pathobiology Johns Hopkins University School of Medicine Baltimore Maryland USA; ^7^ Department of Neurology Johns Hopkins University School of Medicine Baltimore Maryland USA; ^8^ Universidade Federal de São Paulo (UNIFESP); Instituto de Ciências Ambientais, Químicas e Farmacêuticas; Departamento de Ciências Farmacêuticas; Laboratório de Imunologia Celular e Bioquímica de Fungos e Protozoários. Departamento de Ciências Farmacêuticas São Paulo Brazil

**Keywords:** extracellular vesicles, science communication, scientific organizations, social media

## 1 INTRODUCTION

Social media are indispensable for organizations which communicate to a wide target audience. ISEV has been active on social media since it was founded in 2011. As we approach ISEV's 10‐year anniversary on social media platform X, formerly known as Twitter, the members of ISEV's Communications Committee (2022‐2024) evaluated how ISEV has used social media to convey the voice of the Society and its members, as well as looking to the future and how things may change and develop in the years to come.

We hope this editorial inspires you to “connect,” “like,” and “tweet” with us and other EV enthusiasts on social media.

### 1.1 The rise of social media

The rise of social media began about 20 years ago, with the emergence of MySpace (2004), followed by YouTube (2005), Reddit (2005), Facebook (2008), and Twitter/X (2010) (Ortiz‐Ospina, 2019), as well as the more employment‐focused social media platform LinkedIn (2003). Social media have evolved beyond basic communication platforms, by acting as virtual hubs for the exchange of ideas, establishing trends, catalyzing public involvement, maintaining consumer relations, and stimulating brand promotion with a worldwide user base. Furthermore, the seamless integraton of (real‐time) video and photographs enable immersive and engaging interactions between users. The coronavirus pandemic has further increased the number of people active on social media (Dixon, 2022). In a constantly changing world, social media impacts how individuals connect, organizations function, and enterprises navigate the challenges of the modern market.

For scientists, social media enable them to showcase their research, share published papers and preprints, engage in scientific discussions, and connect to peers (Van Noorden, 2014). For ISEV, social media forms important communication routes for people interested in extracellular vesicle (EV) research. Many scientific journals, including those of the International Society of Extracellular Vesicles (ISEV), namely, the Journal of Extracellular Vesicles (JEV) and the Journal of Extracellular Biology (JExBio), use social media to disseminate and promote new articles. Social media were found to be influential in directing researchers to articles published on the websites of scientific journals (Abou‐Ismail et al., 2024). Furthermore, social media coverage directly influences Altmetic attention scores (AAS), which summarize the number of tweets, Facebook posts and blog posts about a publication. Altmetric scores are gaining importance as a metric for the impact of scientific articles, and complement existing metrics such as citation scores. Whether social media coverage/AAS increases long‐term citation scores is still controversial (Özkent, 2022; Sathianathen et al., 2020; Thelwall et al., 2013; Tonia et al., 2020), but in our opinion, sharing one's findings is a logical first step in getting cited. As social media complement traditional routes of science communication (such as papers and conference talks) and are accessible to everybody, social media have become an essential route of science communication whose full impact will unravel in the near future.

**BOX 1 – Facebook profiles, pages and groups: what's the distinction?**

Here we define the differences between these types of sites:

**Facebook profiles** are personal pages that can be used to share information about yourself, as a person or business entity.

**Facebook pages** are business profiles, intended for public figures, brands, organizations to communicate with their fans or customers.

**Facebook groups** are made by people with a Facebook profile, and serve as a hub to talk about shared interests with others.

### 1.2 ISEV's social media presence throughout the years

*Facebook*

ISEV's first step into the realm of social media was taken in January 2011, when the Facebook group “Exosomes, microvesicles and other secreted vesicles” was started by Lawrence Rajendran, during the first “International Workshop on Exosomes” organized in Paris, which also initiated creation of ISEV later that year. This group now has >3,200 members. It began as a Q&A platform, with followers asking and discussing technical questions regarding EV science. Though it is no longer considered an “official” ISEV social media channel, it still serves as a discussion platform for some EV researchers. ISEV decided to move forward with an official page and profile, recognizing and appreciating the efforts of its founding members. In April 2012, the first Facebook group of ISEV was started (https://www.facebook.com/groups/IsevOrg), which now has >2,270 members. ISEV founded a profile page (https://www.facebook.com/internationalsocietyforextracellularvesicles) in September 2016, which is currently followed by > 760 people. These two pages mainly feature announcements from ISEV.

*Twitter/X*

ISEV has two official accounts on Twitter/X, namely @ISEVorg and @ISEV_Journals. ISEV's official society account (@ISEVorg) commenced in December 2014, has > 4,040 followers and is, at the time of publishing, moderated by Tom Driedonks (@tomdriedonks) and Bethany Phipps (Talley Management). These accounts post original content relating to events, publications, interviews with members, information about task forces, special interest groups and, of course, the ISEV annual meeting. The official journal account (@ISEV_Journals) has > 2,900 followers and, at the time of publishing, is moderated by Sarah Williams (@insarahsmind; Executive Scientific Editor at JEV and JExBio). This account disseminates new publications from both ISEV journals; Journal of Extracellular Vesicles (#JEV) and Journal of Extracellular Biology (#JExBio).

Though not an official ISEV account, ISEV also recognizes the work of @ISEVcomms, founded in April 2018, which has > 2,770 followers, and is moderated by Cherie Blenkiron (@cblenkie). This account promotes EV research beyond ISEV's direct efforts, for example, reposting EV publications in non‐ISEV journals and promoting events that are not organized by ISEV. Perhaps in the fullness of time, @ISEVorg and @ISEVcomms will merge, but until such time, we suggest that EV‐enthusiasts on social media keep up with posts from both accounts.

*LinkedIn*

**FIGURE 1:**
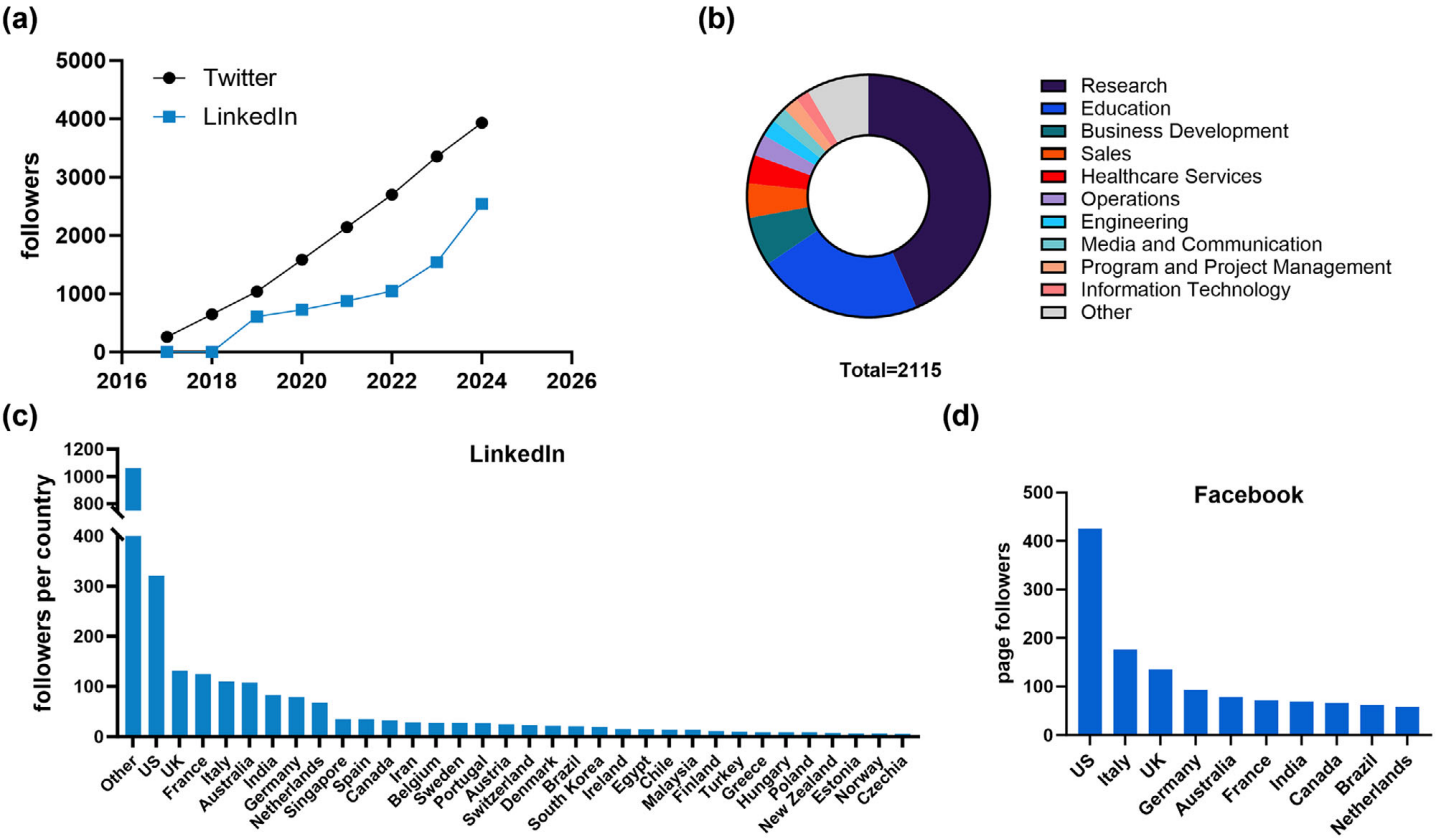
ISEV's social media presence by the numbers. (a) Number of ISEV's followers on Twitter/X (@ISEVorg) and LinkedIn over time. (b) Self‐reported job profiles of ISEV's LinkedIn followers. (c) Geographical distribution of ISEV's LinkedIn followers, by country. (d) Geographical distribution of ISEV's Facebook followers, by country.

ISEV became active on LinkedIn in 2017 (https://www.linkedin.com/company/international‐society‐for‐extracellular‐vesicles/) and has gathered > 2,900 followers to date. Similar to the @ISEVorg Twitter/X account, ISEV's LinkedIn page features event announcements, publications, ISEV member interviews, and news related to task forces and special interest groups. The annual growth of ISEV's Twitter/X (@ISEVorg) and LinkedIn followers are depicted in Figure 1a. ISEV's Twitter/X following has shown constant growth over the years, and ISEV's LinkedIn followers are rapidly catching up.

*Youtube*

In 2020, Kenneth Witwer (@KennethWWitwer) started a YouTube channel to share recordings of the EVClub initiative (https://www.youtube.com/@ExtracellularVesicleClub) which now has > 4,550 subscribers and showcases 243 videos. This channel was originally developed and promoted by Witwer for the benefit of EV scientists and amassed a large number of subscribers. In 2022, ISEV worked with Witwer to make the EVClub channel the official YouTube channel for ISEV. Now, alongside EVClub, this channel increasingly features educational videos made by ISEV, such as recordings of Education Day talks, and ISEV's Massive Open Online Course (MOOC) 3: Detection and Isolation of Intact EVs, as well as recordings of ISEV2023 Featured Abstracts. ISEV hopes that these efforts will help to increase the rigour and reproducibility of science, and to provide educational resources to researchers who may not be able to partake in our in‐person events.

Since 2023, ISEV's Communication Committee has been focusing on harmonizing ISEV's social media output across Twitter/X, Facebook and LinkedIn. ISEV aims to clearly communicate with all of our members, irrespective of their preferred social media platform. In contrast to email announcements, which may be easily overlooked, we hope that our efforts will make our social media platforms work more effectively together and further strengthen the two‐way communication between ISEV's members and the organization.

### 1.3 Global outreach

ISEV's Communication Committee is convinced that social media have the potential to connect a diverse range of people around the world, from different disciplines. To assess where the organization currently stands, we analyzed the job profiles of ISEV's LinkedIn followers (Figure 1b). Approximately two‐thirds of ISEV's followers work in research and education, but also in business development, sales and healthcare. Furthermore, we analyzed the geographical distribution of ISEV's LinkedIn (Figure 1c) and Facebook followers (Figure 1d). Most of ISEV's LinkedIn followers are located in the United States, United Kingdom, and France, and 12 of the top 20 countries are European. Within the Asia‐Pacific chapter, most followers are based in Australia, followed by India, Singapore, Iran and South Korea. Unfortunately, since LinkedIn's “follower analytics” function groups many followers together in an “Other” category, we could not retrieve a complete overview of their geographical distribution. The geographical location of ISEV's Facebook page followers (Figure 1d) shows a large degree of overlap with LinkedIn followers. Brazil and Canada are, however, in the top 10 on Facebook, but not LinkedIn. Twitter/X demographics, unfortunately, could not be analyzed because paid, third‐party applications are required to access this information.

A positive development in the last few years has been the formation of many national societies, which are now part of the Global EV Network (GEVN, https://www.isev.org/national‐societies‐global‐ev‐network). We have overviewed their social media outlets (Table 1) to facilitate engagement with them. From here, it is clear that Twitter/X is used by most societies, followed by Facebook and LinkedIn.

**TABLE 1:** The social media presence of national societies within the Global EV Network.

National society	Twitter/X	Facebook	Linkedin	Instagram
**ASIA‐PACIFIC**				
ANZSEV—Australia and New Zealand Society of Extracellular Vesicles	@ANZSEVresearch	–	–	–
CSEV—Chinese Society for Extracellular Vesicles	–	–	–	–
JSEV—Japanese Society for Extracellular Vesicles	@JSEV5	–	–	–
InSEV—Indian Society for Extracellular Vesicles	@insev_soc	–	https://www.linkedin.com/in/insev/	–
IEVS—Indian Extracellular Vesicles Society	@ISEVSIndia	https://www.facebook.com/groups/698590985564466/	https://www.linkedin.com/company/indian‐extracellular‐vesicles‐society/	–
KSEV—Korean Society for Extracellular Vesicles	–	–	–	–
SOCRATES—Society for Clinical Research and Translation of Extracellular Vesicles Singapore	–	–	–	–
TSEV—Taiwan Society for Extracellular Vesicles	–	https://www.facebook.com/groups/tsevtw	–	–
**AMERICAS**				
ASIC—American Society for Intercellular Communication	@ASICmeeting	–	–	–
CanSEV—Canadian Society for Extracellular Vesicles	@CanVesSociety	–	https://www.linkedin.com/company/canadian‐society‐for‐extracellular‐vesicles/	–
GAVE—Grupo Argentino de Vesículas Extracelulares	@gave_arg	–	–	gave_arg
Latin America Extracellular Vesicles Network	–	https://www.facebook.com/groups/525776894285930	–	–
**EUROPE**				
ASEV—Austrian Society for Extracellular Vesicles (ASEV)	@ASEV_Austria	https://www.facebook.com/ASEVAustria	–	Asev.austria
BSEV—Baltic Society of Extracellular Vesicles	–	https://www.facebook.com/BalticEVsociety	https://www.linkedin.com/company/baltic‐society‐of‐extracellular‐vesicles/	–
BESEV—Belgian Society for Extracellular Vesicles	@BESEV_	–	https://www.linkedin.com/groups/13922264/	–
CzeSEV—Czech Society for Extracellular Vesicles	@CzeSEV	https://www.facebook.com/Czesev.cze/	–	Czesev.cz
DSEV—Danish Society of Extracellular Vesicles	–	–	–	–
ESEV—Egyptian Society of Extracellular Vesicles	–	https://www.facebook.com/profile.php?id=61555719954476	–	–
EVI—Extracellular Vesicles Network of Ireland	–	–	–	–
EVITA—Italian Society of Extracellular Vesicles	@EVItaSociety	–	–	evitasociety
FISEV—Finnish Society of Extracellular Vesicles	@Fisev_society	https://www.facebook.com/groups/161444560964731	–	–
FSEV—French Society of Extracellular Vesicles	@FSEV	–	https://www.linkedin.com/company/french‐society‐for‐extracellular‐vesicles‐fsev/	–
GEIVEX—Grupo Español de Investigación en Vesiculas Extracelulares	@GEIVEX	https://www.facebook.com/groups/1427173657537112	https://www.linkedin.com/groups/12151106/	Geivex_
GSEV—German Society for Extracellular Vesicles	@GSEVorg	https://www.facebook.com/GSEV10	https://www.linkedin.com/company/gsevorg/	Gsev.ev
HSEV—Hungarian Section for Extracellular Vesicles	–	–	–	–
ISREV—Israeli Society for Extracellular Vesicles	@IsrevR	–	–	–
NLSEV—Netherlands Society for Extracellular Vesicles	@NLSEV_	–	https://www.linkedin.com/company/nlsev/	–
NOR‐EV—Norwegian Society for Extracellular Vesicles	@NOR_EVs	–	–	–
PNEV—Portuguese Network on EVs	–	–	–	–
SrbEVs—Serbian Society for Extracellular Vesicles	@SrbEVs_office	https://www.facebook.com/profile.php?id=61556111144561	https://www.linkedin.com/in/serbian‐society‐for‐extracellular‐vesicles‐a13883256/	Srbevs_society
SIN‐EV—Slovenian Network for Extracellular Vesicles	–	–	–	–
SWESEV—Swedish EV Network	@SweEVnet	https://www.linkedin.com/groups/9310373/	–	–
TURSEV—Turkish Society for Extracellular Vesicles	@TURSEV_	–	–	
UKEV—UK Society for Extracellular Vesicles	@UKEVSoc	https://www.facebook.com/groups/2062040314070277/	https://www.linkedin.com/company/united‐kingdom‐society‐for‐extracellular‐vesicles	ukevsoc

Overall, these results indicate that we can improve our outreach in Asia, Africa and Latin America, which ISEV considers an important goal. But how best to do this? ISEV's Communications Committee would appreciate receiving ideas from ISEV members who are located in these geographical areas, as well as other members of the ISEV community. Please reach out to contact@isev.org and the corresponding authors to share your ideas.

### 1.4 Outlook

ISEV has a continuous commitment to enhancing communication and interaction within the global scientific community. To promote more comprehensive geographical representation, ISEV aims to expand its presence in different regions, acknowledging the diversity and importance of varied perspectives in EV research. Furthermore, ISEV will explore novel approaches, such as selecting Twitter/X representatives to promote more live streaming/tweeting during its events. This method will not only work to increase the society's online presence, but also create a more engaging experience for participants and those who cannot attend, enabling the rapid distribution of scientific findings and conversations).

A significant focus is on disseminating scientific advances while also educating and inspiring our members, thereby bridging the gap between the scientific community and the society at large. Young researchers in the EV field are encouraged to partake, with the recognition that they are the society's future. Hosting online events customized to the preferences of each continent is intended to reach a larger audience.

In conclusion, ISEV's uses social media to improve communication, collaboration, and participation within the global scientific community, emphasizing the importance of information exchange and the continuous need for innovation in scientific outreach. The Communications Committee recognizes that social media do not stand still, that content preferences change and new platforms are developed, or old platforms revamped. ISEV currently has good social media coverage, but is not (yet) active on the increasingly popular Instagram or TikTok. Furthermore, ISEV is not reaching all geographical areas equally, on which we would like to do better. ISEV strives to improve its communication with all of its members, and hopes that your comments, interactions, and tweets will guide us in this process. “Follow” us in your own preferred way and make sure you let us know your thoughts.

## AUTHOR CONTRIBUTIONS

**Tom Driedonks**: Conceptualization (lead); data curation (lead); formal analysis (lead); investigation (lead); methodology (lead); project administration (lead); resources (equal); software (equal); supervision (lead); validation (equal); visualization (equal); writing—original draft (lead); writing—review and editing (lead). **Deborah Goberdhan**: Conceptualization (equal); formal analysis (equal); investigation (equal); methodology (equal); project administration (equal); resources (equal); validation (equal); visualization (equal); writing—original draft (equal); writing—review and editing (equal). **Sujata Mohanty**: Conceptualization (equal); data curation (equal); formal analysis (equal); investigation (equal); methodology (equal); resources (equal); software (equal); supervision (equal); validation (equal); visualization (equal); writing—original draft (equal); writing—review and editing (equal). **Sarah Williams**: Conceptualization (equal); data curation (equal); formal analysis (equal); investigation (equal); methodology (equal); resources (equal); software (equal); supervision (equal); validation (equal); visualization (equal); writing—original draft (equal); writing—review and editing (equal). **Rienk Nieuwland**: Data curation (equal); formal analysis (equal); investigation (equal); project administration (equal); resources (equal); software (equal); visualization (equal); writing—original draft (equal); writing—review and editing (equal). **Kenneth Witwer**: Data curation (equal); formal analysis (equal); investigation (equal); methodology (equal); resources (equal); validation (equal); visualization (equal); writing—original draft (equal); writing—review and editing (equal). **Ana Torrecilhas**: Conceptualization (lead); data curation (equal); formal analysis (equal); funding acquisition (lead); investigation (equal); methodology (equal); project administration (equal); resources (equal); software (equal); supervision (lead); validation (equal); visualization (equal); writing—original draft (lead); writing—review and editing (lead).

## CONFLICTS OF INTEREST STATEMENT

The authors declare no conflicts of interest.
